# Electroretinography as a non-invasive biomarker for early detection of cognitive impairment in older adults: A scoping review protocol

**DOI:** 10.1371/journal.pone.0352593

**Published:** 2026-06-26

**Authors:** Isaiah Osei Duah Junior, Katelyn JeNay Houston, Danielle S. Rodriguez, Cassandra M. Germain

**Affiliations:** 1 Department of Optometry and Visual Science, College of Science, Kwame Nkrumah University of Science and Technology, Kumasi, Ghana; 2 Department of Psychology, John R. and Kathy R. Hairston College of Health and Human Sciences, North Carolina Agriculture and Technical State University, Greensboro, North Carolina, United States of America; Tehran University of Medical Sciences, IRAN, ISLAMIC REPUBLIC OF

## Abstract

Cognitive decline and dementia represent a growing global health challenge with an urgent need for accessible biomarkers to support early detection, monitoring, and timely intervention, particularly among marginalized populations who face persistent barriers to equitable access to specialized diagnostic and neurological care. Electroretinography (ERG), a non-invasive measure of retinal electrophysiological activity, has emerged as a potential visual biomarker given the retina’s structural and functional homology with the brain. Preliminary studies suggest associations between ERG abnormalities and cognitive impairment; however, the existing evidence base is limited and lacks sufficient consistency and integration to draw clear, definitive conclusions. The primary aim of this scoping review is to systematically characterize and synthesize existing evidence on the utility and efficacy of ERG as a visual biomarker of cognitive change and cognitive status in adults 50 years and over and identify research gaps. Specifically, the review will: (1) characterize study designs, populations, ERG modalities, parameters, and cognitive outcomes; (2) identify ERG components consistently associated with cognitive status or decline; (3) describe strategies for managing ophthalmic and systemic confounders; (4) locate longitudinal studies assessing prognostic or predictive utility; and (5) highlight methodological limitations, reporting gaps, and priority areas for future research. Following the Population–Concept–Context (PCC) framework, the review will include studies of older adults with cognitive impairment, or dementia, examining ERG modalities such as full-field, pattern, and multifocal ERG. All relevant and completed published studies in English language will be identified from MEDLINE (via PubMed), Embase, Web of Science, Scopus, PsycINFO, Google Scholar, Cochrane Central Register of Controlled Trials (CENTRAL) without date restrictions. The retrieved records will be managed in EndNote (version 2025), and in accordance with Covidence framework for scoping reviews, three reviewers will independently screen titles, abstracts, and full texts. Data will be charted on study characteristics, ERG parameters, cognitive outcomes, and ERG-cognitive status associations. Collectively, this synthesis will provide clinicians and researchers with consolidated evidence on ERG–cognition relationships, clarify whether the evidence base supports further systematic review or meta-analysis, and generate methodological recommendations for future studies.

## Introduction

Globally, unprecedented population ageing, combined with increased life expectancy [1,2], socioeconomic transitions, and increases in cardiometabolic diseases [3–5], have led to a surge in neurodegenerative and neuropsychiatric disorders, accompanied by progressive cognitive and memory decline [1–5]. These conditions, together with their associated risk factors, are becoming increasingly prevalent and contribute substantially to the rising burden of cognitive impairment worldwide [6]. Current estimates indicate that between 32 and 315 million individuals fall along the Alzheimer’s disease pathology continuum [7], while the global prevalence of dementia is projected to rise sharply from 57.4 million cases in 2019 to 152.8 million by 2050 [8]. At the individual level, these projections underscore the urgent need for early identification of populations at risk of cognitive decline, to enable effective risk stratification and timely clinical intervention [9]. At the societal level, they highlight the necessity of informed public health planning, strategic resource allocation, and investment in innovative therapeutic approaches aimed at slowing disease progression and mitigating future burden [8].

The rapid expansion of dementia research, coupled with advances in biomedical technologies, has facilitated the development of several brain-based biomarkers with significant diagnostic and prognostic utility [10]. These include neuroimaging modalities [11], cerebrospinal fluid assays [12,13], and blood-based markers [12,13]. Despite their clinical value, these approaches often require specialized infrastructure, may be invasive or costly, and remain inaccessible in many clinical and low-resource community settings [14–16]. These factors underscore the need for non-invasive, cost-effective, and consumer-friendly technologies to support the timely detection and effective management of cognitive decline [17].

The human retina offers a unique and highly accessible window into central nervous system (CNS) pathology [17–19]. As an embryological outgrowth of the brain, the retina is part of the CNS and shares key structural and functional properties with cortical tissue [18,20]. To this end, retinal function can be assessed rapidly and non-invasively, making it particularly suitable for large-scale community-based screening and longitudinal monitoring of cognitive dysfunction in adults, including geriatric populations and marginalized groups with limited access to neurological care. Of note, the electroretinogram provides an objective measure of retinal electrophysiological function by capturing signal transduction across photoreceptors, bipolar cells, ganglion cells, and inner retinal circuits [21]. Because these retinal elements mirror core features of central synaptic and neural network organization, the ERG serves as a functional readout of broader brain integrity [17,22–24].

Consistent with this notion, ERG abnormalities have been demonstrated across diverse neuropsychiatric and neurodegenerative conditions, supporting its role as a functional proxy for brain integrity and cognitive health [17,22,23,25–32]. By detecting subtle retinal dysfunction that may precede overt clinical symptoms, ERG represents a promising biomarker for memory deficits, cognitive decline, and dementia-related pathophysiology [16,17,33]. Taken together, ERG holds potential for early risk stratification, improved disease monitoring, and the identification of individuals who may benefit from timely preventive or therapeutic interventions.

### The rationale for conducting the scoping review

The growing interest in the retina–brain connection has prompted increasing investigation into whether specific ERG parameters including a-wave and b-wave amplitudes, implicit times, and oscillatory potentials across full-field ERG (ffERG), pattern ERG (pERG), and multifocal ERG (mfERG) are altered in persons living with MCI, Alzheimer’s disease [27,30,32,34–42], and other neuropsychiatric disorders compared with cognitively intact adults [31,43–51]. Preclinical and clinical studies suggest meaningful associations between cognitive dysfunction and ERG abnormalities, including reduced amplitudes, delayed implicit times, and altered inner-retinal oscillatory activity [36,52,53]. Together, these findings raise the possibility that ERG could serve as a low-cost, widely deployable ocular biomarker to complement and/or enhance existing diagnostic testing paradigm for cognitive deficits [16]. However, the evidence base remains fragmented, varied in quality, and methodologically inconsistent, making it difficult to draw firm conclusions about ERG’s utility in cognitive-health assessment.

Further, studies differ substantially in the ERG modalities used, stimulus protocols, retinal ERG components analyzed (e.g., a-wave, b-wave, implicit times, oscillatory potentials), and the cognitive outcomes measured [16,17,42,35,54–57]. Additional variability arises from differences in diagnostic criteria used for neurocognitive disorder, sample characteristics, study designs, and the handling of key confounders such as age-related ocular disease, systemic vascular factors, medication effects, and technical differences in ERG acquisition [27,30,32,34–46,58,59]. Importantly, recent advances have introduced portable ERG device mainly RETeval that enable rapid, non-invasive assessment outside of specialized laboratory settings [60–62]. These innovations have the potential to expand ERG feasibility as a scalable, community-based screening platform, by reducing barriers related to cost, accessibility, and infrastructure [60,61]. The deployment of portable ERG technologies in primary care, community clinics, and population-level studies will help bridge the gap between experimental promise and practical implementation in cognitive-health assessment.

The fragmentation of existing evidence has resulted in a landscape in which findings are difficult to synthesize, and the conceptual boundaries of the field remain unclear. Some studies examine ERG as a diagnostic tool, others as a method for disease staging, and still others as a means of monitoring cognitive decline over time [16,37,54,63]. Many investigations are small-scale, exploratory, or pilot in nature, and methodological rigor varies widely [16,17,42,35,54–57,64–67]. Collectively, these limitations have hindered the development of coherent conclusions regarding the role of ERG in cognitive impairment and dementia among older adults. Despite growing interest, there remains a lack of comprehensive evidence mapping that systematically links specific ERG measures with trajectories of cognitive decline.

To address this knowledge gap, a scoping review represents the most appropriate methodological approach [68–70]. Unlike traditional systematic reviews or meta-analyses, which typically focus on narrowly defined questions and relatively homogeneous data, scoping reviews are designed to map broad and evolving fields of inquiry [68–70]. This approach allows for the inclusion of diverse study designs and enables systematic identification of the ERG parameters assessed, clarification of how cognitive impairment is operationalized, and evaluation of variability in study methodologies and reporting practices [68–70]. A scoping review can also illuminate critical evidence gaps relevant to our hypotheses, including the need for longitudinal studies, the lack of standardized ERG acquisition and analysis protocols, and limited consideration of comorbid ocular or systemic conditions that may influence retinal signals [68–70].

By systematically charting the extent, range, and nature of research on ERG and cognitive impairment, this review will provide a foundational understanding of ERG’s potential utility in applied psychology and, more specifically, in aging neuroscience [71]. It will support researchers, clinicians, and policymakers in identifying priorities for future investigations, inform the development of standardized research frameworks, and determine whether subsequent systematic reviews or meta-analyses are feasible. Collectively, this synthesis will clarify whether ERG can be positioned as a credible, scalable ocular biomarker for cognitive decline in gerontology, complementing existing diagnostic approaches and advancing both research and clinical practice.

### The aim of the scoping review

The primary aim of this scoping review is to systematically map existing evidence on the utility and efficacy of ERG as an ocular biomarker for cognitive changes in older adults (≥ 50 years), with specific attention to its relationships with cognitive impairment and dementia. This review will describe and categorize ERG modalities including ffERG, pERG, mfERG as well as the recording protocols used across studies, such as ERG system type, recording protocols, stimulus properties. It will also summarize the retinal ERG measures most evaluated, including amplitudes, implicit times, and waveform characteristics (specific aim 1). The review will further identify the cognitive assessment approaches used in studies examining ERG in relations to cognition, including the cognitive domains assessed, the neuropsychological tests employed, and the diagnostic criteria used to classify cognitive status (specific aim 2). In addition, it will examine methodological characteristics and sources of heterogeneity across studies, including study design, sample composition, strategies for handling ocular comorbidities confounds, and differences in ERG acquisition and analysis (specific aim 3). The synthesis will examine how alterations in ERG parameters including amplitudes, latencies, oscillatory potentials and the photopic negative response are associated with performance on cognitive tasks and overall cognitive status. It will consider both cross sectional findings and longitudinal or predictive evidence reported in the literature (specific aim 4). Finally, the review will identify research gaps, methodological inconsistencies and limitations in the existing evidence base to guide future research, particularly regarding feasibility, standardization requirements and the potential clinical utility of ERG. It will also map how ERG has been integrated with other biomarkers such as neuroimaging, structural retinal imaging, and fluid-based markers in studies examining cognitive decline (specific aim 5).

## Methods

**Fig 1:**
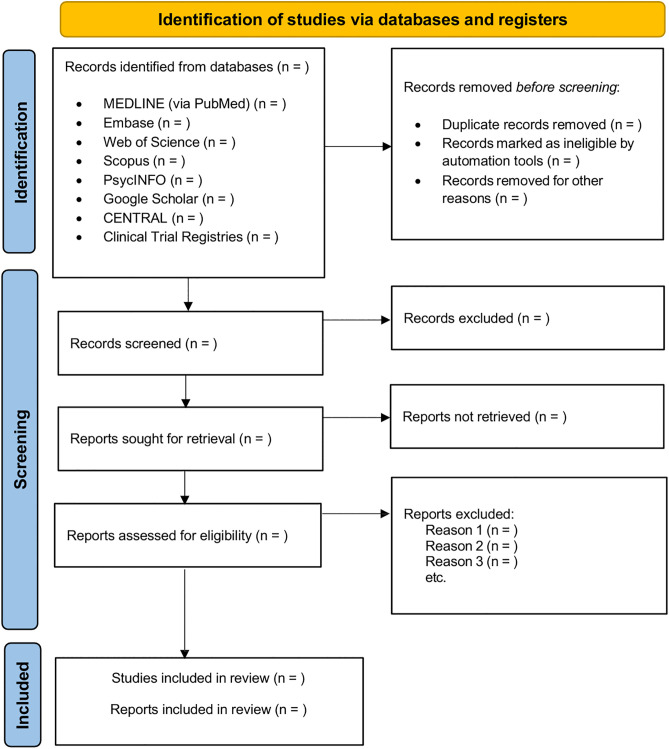
PRISMA flow diagram. This chart delineates the step-by-step process of how data will be charted.

The scoping review protocol was prepared in accordance with PRISMA-P (S1 Table) [72] and indexed in the Open Science Framework (10.17605/OSF.IO/PM8XC) to ensure methodological rigor and transparent reporting. This protocol outlines a structured framework designed to identify and synthesize evidence on the relationship between ERG and cognitive status, as illustrated in the PRISMA flow diagram (see Fig 1). The review will follow the PCC framework to guide study identification and selection [73] and systematically chart the emerging findings in alignment with established scoping review methodology proposed by Arksey and O’Malley [71]. Specifically, the review will adopt five-stage process which includes: (1) identifying the research question, (2) identifying relevant studies, (3) selecting studies, (4) charting the data, and (5) collating, summarizing, and reporting the results [71]. This structured approach provides a transparent and replicable framework for mapping evidence across diverse study designs of cognitive assessment in aging adults. The final review will be reported in accordance with the PRISMA-ScR to maintain comprehensive reporting standards and methodological rigor [74]. Collectively, this scoping review protocol outlines a framework for systematically mapping how ERG has been investigated in relation to cognitive status, clarifying the current evidence base, identifying methodological strengths and research gaps, and informing future research and clinical translation in geriatric care.

### Inclusion criteria

#### Population.

This scoping review will include studies involving adult populations aged 50 years and older across the cognitive spectrum, including cognitively intact individuals, those at risk of cognitive decline, and individuals diagnosed with MCI and Alzheimer’s disease and -related dementia (ADRD). Eligible studies will consist of human research investigations using randomized controlled trial (RCT), quasi-experimental, and observational research designs. Studies will be included participants’ cognitive status has been assessed using validated neuropsychological instruments or standardized cognitive screening toolkits such as Mini-Mental State Examination (MMSE) [75,76], Montreal Cognitive Assessment (MoCA) [77,78], Cambridge Neuropsychological Test Automated Battery (CANTAB) [79], Benton Visual Retention Test (BVRT) [80], Wechsler Memory Scale (WMS) [81]. Studies will also be eligible if cognitive status is defined using established diagnostic criteria, including the Diagnostic and Statistical Manual of Mental Disorders (DSM) [82,83], the International Classification of Diseases (ICD) [84], or the National Institute on Aging–Alzheimer’s Association (NIA‑AA) framework [85].

#### Concept.

**Fig 2:**
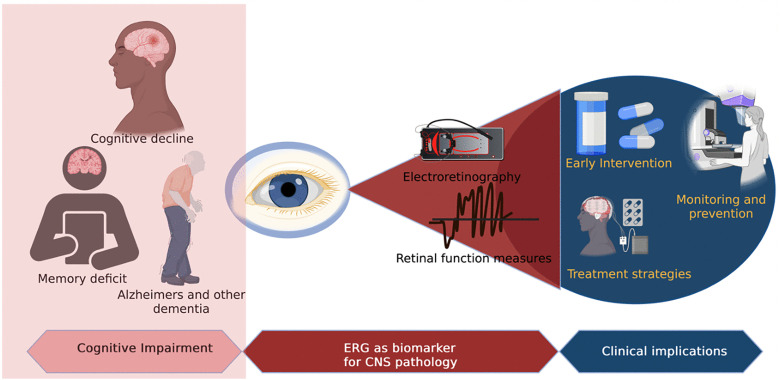
ERG as a biomarker for cognitive decline and CNS pathology. This schematic illustrates the potential role of electroretinography (ERG) in detecting cognitive impairment and neurodegenerative conditions such as Alzheimer’s disease and other dementias. ERG measures retinal function, capturing electrophysiological signals that reflect central nervous system (CNS) integrity. Alterations in ERG parameters may serve as early indicators of cognitive decline, enabling timely intervention, monitoring, and preventive or therapeutic strategies. The figure highlights the translational pathway from retinal electrophysiology to clinical application, emphasizing ERG as a scalable biomarker bridging ocular measures and CNS pathology. Created in BioRender. Osei Duah, **I. J.** (2026) https://BioRender.com/f4fqajq.

The core concept of this scoping review is the use of ERG as a biomarker of cognitive decline and cognitive status. Eligible studies may employ any ERG modality, including ffERG**,** pERG**,** and mfERG and must report quantitative or qualitative retinal functional parameters such as amplitudes, implicit (latency) time, and other waveform morphology. Studies will be included if ERG findings are examined in relation to cognitive performance, cognitive trajectories, or neurodegenerative processes associated with cognitive impairment or ADRD (see Fig 2)**.**

#### Context.

This scoping review will encompass evidence generated across a broad range of research and care settings, including hospital-based studies, outpatient and specialty clinics, community-based cohorts, and population-level or epidemiological investigations. No geographical restrictions will be applied, allowing inclusion of studies from both high- and low-resource settings. Studies published in any language will be considered, provided that an English abstract is available to support screening and eligibility assessment. Both RCT, quasi-experimental, and prospective research designs will be included to comprehensively map how ERG has been applied to evaluate cognitive status, detect early cognitive changes, and monitor cognitive decline over time.

### Exclusion criteria

Studies focusing exclusively on pediatric, adolescent, or young adult populations will be excluded. Studies using ERG solely to assess primary ophthalmic disorders (e.g., glaucoma, age-related macular degeneration, rod–cone dystrophies) will be excluded unless they explicitly report cognitive outcomes or examine associations between ERG parameters and cognitive status. *In vivo* animal studies, *in vitro* studies, case reports, editorials, commentaries, conference abstracts, review articles, and non–peer-reviewed publications will be excluded to ensure inclusion of rigorously evaluated and methodologically sound evidence. Articles without accessible full text will also be excluded due to insufficient detail for critical appraisal. Additionally, studies lacking validated cognitive assessments or clear diagnostic criteria for cognitive status, or those with inadequately described ERG methods or cognitive measures that are not analytically linked, will be excluded to maintain methodological rigor and interpretability.

### Data sources

A comprehensive and systematic search will be conducted across major bibliographic databases to capture peer-reviewed literature relevant to the use of ERG in cognitive-health research. Databases will include MEDLINE (via PubMed), Embase, Web of Science, Scopus, PsycINFO, Google Scholar, and the CENTRAL, to ensure broad coverage across biomedical, psychological, and interdisciplinary domains. Citation searching will be performed on included studies to identify additional relevant publications. The search will be restricted to peer-reviewed published studies to ensure methodological rigor, data reliability, and consistency in quality appraisal. The review aims to exclude unpublished and grey literature sources to enhance the credibility and reproducibility of findings. No date or language restrictions will be applied, and eligible non-English studies will be translated where necessary to ensure consistent reporting.

### Search strategy

Comprehensive searches will be conducted across all selected databases, with search strategies developed in consultation with a medical librarian to ensure methodological rigor and reproducibility. The search framework will be organized around three core concepts (see Table 1 for detailed terms and strategy). Boolean operators will be used to structure the search, applying “OR” to combine synonyms within each concept and “AND” to link distinct concepts. Search syntax will be adapted to ensure compatibility across databases. Controlled vocabulary, including medical subject headings (MeSH) and Emtree terms, will be integrated with free-text keywords related to ERG and cognitive outcomes. Proximity operators and database-specific filters will be applied where appropriate to optimize sensitivity and precision. All search strings, database interfaces, and search dates will be systematically documented and reported in accordance with PRISMA-ScR guidelines [74].

**Table 1:** PCC-based search strategy.

Serial No.	PCC Element	Search Terms (OR-separated)
#1	Population	cognitive decline OR cognitive impairment OR cognitive dysfunction OR cognitive deterioration OR cognitive deficit OR cognitive disorder OR neurocognitive decline OR neurocognitive impairment OR mild cognitive impairment OR MCI OR dementia OR Alzheimer disease OR neurodegenerative disease OR neuropsychiatric disorder
#2	Concept	electroretinography OR electroretinogram OR ERG OR retinal electrophysiology OR retinal electrical response OR retinal function test
#3	Context	clinical setting OR healthcare setting OR hospital OR outpatient OR community-based OR research setting
#4	Combined PCC Search (optional)	(electroretinography OR electroretinogram OR ERG OR retinal electrophysiology OR retinal electrical response OR retinal function test) AND (cognitive decline OR cognitive impairment OR cognitive dysfunction OR cognitive deterioration OR cognitive deficit OR cognitive disorder OR neurocognitive decline OR neurocognitive impairment OR mild cognitive impairment OR MCI OR dementia OR Alzheimer disease OR neurodegenerative disease OR neuropsychiatric disorder) AND (clinical setting OR healthcare setting OR hospital OR outpatient OR community-based OR research setting)

Boolean operators: “OR” and “AND”.

### Study selection

All retrieved records will be managed in EndNote (version 2025) and subsequently imported into Covidence for duplicate removal and screening. Study selection will follow a two-stage process in accordance with PRISMA-ScR guidelines. First, titles and abstracts will be screened for relevance. Full text of potentially eligible studies will then be reviewed against the prespecified inclusion criteria. Each record will be independently assessed by two reviewers to minimize selection bias. Discrepancies will be resolved through discussion and majority voting (2:1). If consensus cannot be achieved, an independent reviewer will adjudicate. The study selection process will be documented using a PRISMA flow diagram. When necessary, corresponding authors will be contacted to clarify unclear or missing information.

### Data extraction

Three authors will independently extract data from each included study using a piloted data extraction form adapted from the Joanna Briggs Institute data collection form (see Table 2). The ERG parameters will be treated as the primary predictors and will include ERG waveform components such as amplitudes and latencies, oscillatory potentials, the photopic negative response (PhNR), ERG modality type, and derived indices of retinal function from other modalities. The primary outcomes will consist of cognitive status and performance, operationalized through clinical classifications, for example cognitively intact, MCI, or dementia, as well as global cognitive screening scores and domain-specific measures of memory, executive function, attention, and processing speed. Secondary outcomes will include disease stage, symptom severity, and longitudinal cognitive trajectories where reported. Data extraction will also capture potential modifiers of ERG–cognition associations, including participant characteristics such as age, sex, and education; visual health confounders including visual acuity, cataract, glaucoma, and retinal pathology; and ERG methodological features. The extent to which ERG findings are integrated with other biomarkers, such as neuroimaging, cerebrospinal fluid indices, or blood-based measures, will be documented. Methodological characteristics, study limitations, and reporting quality will be systematically charted to delineate sources of heterogeneity, synthesize key findings, and identify evidence gaps and priorities for future investigation (see Table 2).

**Table 2:** Data extraction framework for ERG–cognition studies.

Serial No.	Variable	Description of the Variable
1	Study identification	Author(s), year of publication, country/region, source type, study objectives/aim, funding, conflicts of interest
2	Study design	Study design, sample size, study setting, inclusion/exclusion criteria, follow-up duration, statistical methods
3	Participant characteristics	Age, sex distribution, education, cognitive status, diagnostic criteria, medical comorbidities, ocular health/visual acuity
4	Ocular and systemic confounders	Screening for ocular diseases and systemic conditions that affects the retina, exclusion or adjustment methods, refractive error, pupil dilation control
5	ERG characteristics	ERG modality, recording protocol, stimulus characteristics, retinal parameters, retinal cell/pathway target
6	Cognitive assessment	Cognitive domains assessed, tests/tools used, cut-off scores, global versus domain-specific measures
7	ERG–cognitive associations	Association direction, ERG parameters linked, cognitive domains affected, type of evidence
8	Integration with other biomarkers	Neuroimaging, retinal imaging, blood/fluid biomarkers, multimodal analysis
9	Methodological characteristics and limitations	ERG protocol variability, cognitive classification differences, sample heterogeneity, confounding factors, study limitations
10	Key findings and evidence gaps	Main findings, evidence gaps, future research recommendations, clinical/feasibility implications

ERG, electroretinogram.

### Data synthesis

Data synthesis will follow established methodological guidance for scoping reviews and will be reported in accordance with the Synthesis Without Meta-analysis (SWiM) framework which is appropriate when statistical pooling is not feasible due to heterogeneity [86]. Because the included studies are expected to differ substantially in ERG modalities, cognitive assessments, study designs, and analytic approaches, no meta‑analytic effect estimates will be generated. Instead, the synthesis will rely on structured, transparent comparison methods suited for diverse evidence. Following data charting, studies will be organized according to ERG modality (ffERG, pERG, and mfERG), waveform morphology (a-wave and b-wave amplitudes, implicit times, oscillatory potential, PhNR etc.), population characteristics (cognitively intact, MCI, and ADRD), and study design (RCT, quasi-experimental, and observational designs). Reported ERG–cognitive associations will be categorized by direction of effect (positive, negative, null, or mixed), statistical significance, and effect size metrics where available (e.g., correlation or regression coefficients, odds ratios, and 95% confidence intervals). A structured narrative synthesis will compare patterns of association across studies, emphasizing convergence, inconsistency, and sources of variation. Quantitative findings will be tabulated and visually mapped to facilitate comparison across ERG parameters and cognitive domains, including global cognition, memory, executive function, attention, and processing speed. Longitudinal evidence will be synthesized separately to evaluate whether specific ERG parameters demonstrate predictive utility for cognitive decline or disease progression. Methodological characteristics likely to contribute to heterogeneity will be examined systematically, including ERG acquisition protocols (stimulus conditions, adaptation state, electrode configuration), cognitive assessment instruments, diagnostic criteria, sample size, adjustment for confounders (age, sex, education), and control of ophthalmic or systemic comorbidities. These factors will inform interpretation of the strength and consistency of reported associations. Although no pooled effect estimates will be produced, the review will assess whether any subgroup of studies demonstrates sufficient methodological and outcome homogeneity to justify a future meta‑analysis. The resulting SWiM‑informed synthesis will provide a transparent and reproducible summary of the extent, nature, and methodological robustness of the evidence base, while identifying priorities for harmonization and future investigation [86].

### Ethical consideration

This scoping review protocol is based entirely on previously published studies and secondary data sources. No new data will be collected from human participants or animals, and no interventions or experimental procedures will be conducted; therefore, ethical approval is not required. The protocol will follow established standards of research conduct, including transparent reporting, accurate citation of original sources, and responsible synthesis of evidence. In accordance with the PLOS Data Policy, all data underlying the findings of the completed scoping review will be made fully available at the time of publication. This will include the complete set of extracted variables, data-charting tables, and any summary statistics derived from included studies. If any included studies impose limitations on sharing specific data elements because of third-party ownership or licensing constraints, these restrictions will be clearly documented, and only data that can be shared legally and ethically will be made available. All included studies will be appropriately referenced to respect intellectual property and maintain the integrity of the original research.

## Discussion

This scoping review protocol outlines a systematic approach to map and synthesize existing evidence on the use of ERG as an ocular biomarker for cognitive changes or cognitive status in older adults. By cataloguing ERG indices and modalities, cognitive domains, assessment tools, and diagnostic classifications across studies, the review aims to provide a comprehensive overview of how ERG has been applied in research on cognitive impairment and dementia in the aging populations. The synthesis will examine reported associations between ERG measures and cognitive outcomes, capturing both cross-sectional relationships and longitudinal or predictive patterns. In doing so, the review will delineate the scope, depth, and characteristics of the current literature while highlighting areas where evidence is emerging or remains limited.

Several methodological considerations are anticipated. Substantial heterogeneity in study designs, participant populations, ERG acquisition protocols, and approaches to managing ocular comorbidities is expected, which will preclude quantitative pooling and necessitate a narrative synthesis. This heterogeneity will also limit direct comparability across studies. As a scoping review, formal assessment of risk of bias or strength of evidence will not be conducted, and conclusions regarding clinical effectiveness or diagnostic accuracy will remain cautious. Nonetheless, by systematically identifying methodological variability, evidence gaps, and inconsistencies, the review is expected to inform future primary studies and systematic reviews, support efforts toward methodological standardization. It will also guide the development of rigorous longitudinal and multimodal investigations of ERG as a biomarker of cognitive decline among aging population.

## Supporting information

S1 Table PRISMA-P (Preferred Reporting Items for Systematic Review and Meta-Analysis Protocols) 2015 checklist: Recommended items to address in a systematic review protocol. (DOCX)

## Abbreviations

ADRD: Alzheimer’s Diseases and Related Dementia
BVRT: Benton Visual Retention Test
CANTAB: Cambridge Neuropsychological Test Automated Battery
CENTRAL: Cochrane Central Register of Controlled Trials
DSM: Diagnostic and Statistical Manual of Mental Disorders
ERG: Electroretinography
ffERG: Full-field ERG
JBI: Joanna Briggs Institute
MCI: Mild Cognitive Impairment
mfERG: Multifocal ERG
MMSE: Mini-Mental State Examination
MoCA: Montreal Cognitive Assessment
PCC: Population–Concept–Context
pERG: Pattern ERG
PRISMA: Preferred Reporting Items for Systematic Review and Meta-analysis
PRISMA-ScR: Preferred Reporting Items for Systematic Review and Meta-analysis Extension for Scoping Reviews
WMS: Wechsler Memory Scale
